# IL-1β receptor antagonist (IL-1Ra) combined with autophagy inducer (TAT-Beclin1) is an effective alternative for attenuating extracellular matrix degradation in rat and human osteoarthritis chondrocytes

**DOI:** 10.1186/s13075-019-1952-5

**Published:** 2019-07-10

**Authors:** Fen Wang, Jijie Liu, Xiaolei Chen, Xinpeng Zheng, Ning Qu, Bing Zhang, Chun Xia

**Affiliations:** 1 Zhongshan Hospital, Xiamen University, Xiamen, 361004 Fujian China; 2 School of Medicine, Xiamen University, Xiamen, 361102 Fujian China

**Keywords:** IL-1β, IL-1Ra, Autophagy, ECM, OA chondrocytes, Rat OA model

## Abstract

**Background:**

Autophagy induction is an effective approach for OA therapy. IL-1β is one of the major inflammatory cytokines linked to OA pathological progression, and its receptor blockade interrupts OA cartilage destruction. The objective of this study was to decipher the link between autophagy and regulatory mechanism of IL-1β and to investigate the effect of IL-1β receptor blockade by IL-1 receptor antagonist (IL-1Ra) combined with or without an autophagy inducer (TAT-Beclin1) on extracellular matrix (ECM) in OA chondrocytes in vitro *and* in vivo.

**Methods:**

IL-1β-treated rat and human OA chondrocytes were cultured in response to IL-1Ra. The expression and distribution of signal molecules regulating ECM synthesis and autophagy were investigated via western blotting, immunoprecipitation, real-time PCR, immunofluorescence, and transmission electron microscope technique. Furthermore, after intra-articular injection of IL-1Ra, TAT-Beclin1, and a combination of both in a rat OA model established by anterior cruciate ligament transection and medial meniscus resection, the morphological changes of cartilage and related signal molecule expression levels were monitored using H.E., Safranin O-Fast green, and immunohistochemistry staining.

**Results:**

Reduced autophagy by IL-1β contributed to ECM degradation, and blockade of IL-1β by IL-1Ra restored autophagy and attenuated ECM degradation in rat and human OA chondrocytes, as well as in a rat OA model. Akt/mTOR/ULK1, Akt/mTOR/NF-κB, and LC3B deacetylation were involved in autophagy regulated by IL-1β. Intra-articular injection of IL-1Ra combined with TAT-Beclin1 was more effective than IL-1Ra alone.

**Conclusions:**

IL-1Ra restored autophagy and attenuated ECM degradation, with an implication that blocking IL-1β combined with enhancing autophagy might be a potential therapeutic strategy for OA.

**Electronic supplementary material:**

The online version of this article (10.1186/s13075-019-1952-5) contains supplementary material, which is available to authorized users.

## Background

Osteoarthritis (OA) pathological progression is linked to some cytokines, such as interleukin-1β (IL-1β) and tumor necrosis factor alpha. IL-1β is produced not only by activated synoviocytes and mononuclear cells, but also by articular cartilage chondrocytes [1]. It could significantly upregulate metalloproteinase (MMP) gene expression, resulting in extracellular matrix (ECM) degradation [2]. Blocking IL-1β, including recombinant IL-1 receptor antagonists (IL-1Ra) or soluble IL-1 receptor proteins, could modify OA progression [2]. Especially, elevated reproductively IL-1Ra to block IL-1β is a crucial element to promote cartilage regeneration in Orthokine®, which is an effective and well-tolerated alternative to currently predominant treatments of OA [3]. However, the mechanism of blocking IL-1β by IL-1Ra to promote cartilage regeneration is not fully understood [4].

As a highly conserved homeostatic process, autophagy could sequester and degrade cytosolic macromolecules, excess, or damaged organelles, and some pathogens to maintain cellular homeostasis in a healthy condition. Autophagy induction could interrupt OA pathological progression, including chondrocyte survival and ECM metabolism, and be an effective approach for OA therapy [5–9]. For instance, autophagy is a protective mechanism in normal cartilage [6]. The enhancement of autophagy by intra-articular injection of mTOR inhibitor, Rapamycin or Torin1, reduces degeneration of articular cartilage in an animal OA model [7–9]. However, whether and how IL-1β in OA progression linked to autophagy is not carefully elucidated. It is previously reported that autophagy regulates the secretion of IL-1β in macrophages [10–12]. Autophagy controls the production of IL-1β in macrophage through at least two separate mechanisms: by targeting pro-IL-1β for lysosomal degradation and by regulating activation of the NLRP3 inflammasome [10]. The inhibition of autophagy in macrophages by knockdown of autophagy-related gene (Atg)7 or Atg16L1 promotes the secretion of IL-1β in response to LPS [11]. On the other hand, IL-1β could modify autophagy in macrophage. IL-1 receptor blockade restores autophagy of macrophage and reduces inflammation in chronic granulomatous disease in mice and in humans [12]. Based on these studies, we hypothesize that the regulatory effect of IL-1β in OA progression might be associated with autophagy.

In this study, it was investigated the relationship between ECM synthesis and autophagy in IL-1β-treated rat chondrocytes (mimicking OA pathological condition, hereafter referred to as OA chondrocyte) and human OA chondrocytes in response to IL-1Ra. Furthermore, the effects of IL-1Ra on articular cartilage were monitored in a rat OA model. Our results demonstrated that reduced autophagy contributed to ECM degradation by IL-1β and blockade of IL-1β by IL-1Ra restored autophagy and attenuated ECM degradation in rat and human OA chondrocytes, as well as in a rat OA model. Akt/mTOR/ULK1, Akt/mTOR/NF-κB, and LC3B deacetylation were involved in autophagy regulated by IL-1β. Intra-articular injection of IL-1Ra combined with autophagy inducer (TAT-Beclin1) is an effective alternative for attenuating extracellular matrix degradation in a rat OA model.

## Methods

### Reagents and antibodies

Antibodies against mTOR (1:1000), p-mTOR (Ser2448) (1:1000), p-ULK1 (S757) (1:1000), Akt (1:1000), p-Akt (S473) (1:1000), NF-κB(p65) (1:1000), p-p65 (S536) (1:1000), acetylated lysine (Ace-lys) (1:1000), and Beclin1(1:1000) were purchased from Cell Signaling Technology Inc. (Beverly, MA, USA). Antibodies targeting Col II (1:1000 for western blotting analysis, 1:200 for immunohistochemistry staining), ULK1 (1:1000), and p62 (1:1000) were purchased from Abcam Inc. (Cambridge, MA, USA). Antibodies against Aggrecan (1:1000 for western blotting analysis, 1:200 for immunohistochemistry staining) and β-actin (1:40,000) were purchased from Sigma-Aldrich in China (Shanghai, China). Rabbit IgG and Protein A/G PLUS-Agarose were purchased from Santa Cruz Biotechnology (Santa Cruz, CA, USA). Antibodies against LC3B (1:1000 for western blotting analysis, 1:200 for immunohistochemistry staining), Lamin B1 (1:1000), and β-tubulin (1:1000) were purchased from Novus Biologicals, Inc. (Littleton, CO, USA), Protein Tech Group (Chicago, IL, USA), and Sangon Biotech (Shanghai, China), respectively. Other reagents were of the highest grade commercially available.

### Chondrocyte isolation, culture, and treatment with IL-1β

After the approval of the Committee on the Ethics of Animal Experiments of Medical School, Xiamen University, rat chondrocytes of knee cartilage were isolated from neonatal male Sprague–Dawley (SD) rats (within 24–72 h after birth) [13, 14]. Briefly, rats were killed and articular cartilages were removed under sterile conditions. Thin slices of cartilage were sequentially digested with 0.25% Trypsin 37 °C for 5 min, followed with 0.1% type I collagenase (Sigma-Aldrich in Shanghai, China) in humidified incubator (37 °C and 5% CO_2_) overnight. Type I collagenase was prepared with Dulbecco’s modified Eagle’s medium (DMEM)/F12 (Invitrogen, Carlsbad, CA, USA) containing 10% FBS supplemented with antibiotics: penicillin (100 UI/ml, Sigma) and erythromycin (100 μg/ml). Primary chondrocytes were cultured in DMEM containing 10% fetal bovine serum to 80% confluence and plated in 60-mm cell culture dishes (1 × 10^6^/ml). Passage 1–2 chondrocytes obtained from the same rat in each experiment were pretreated by IL-1β (20 ng/ml) for 36 h to mimic OA pathological condition for the subsequent experiments [14].

Human OA cartilage was obtained from 27 patients (Table 1) with advanced OA who were undergoing total knee replacement surgery without pro-treatment of arthroscopy, after receiving all patient consent and in accordance with the ethical guidelines approved by the Ethics Committee of Medical School, Xiamen University, China. Thin slices of cartilage were sequentially digested with 0.25% trypsin 37 °C for 30 min and 0.15% type II collagenase (Sigma-Aldrich in Shanghai, China) in a humidified incubator (37 °C and 5% CO_2_) for 8–12 h. Type II collagenase preparation is the same as type I collagenase. Primary chondrocytes were cultured in DMEM containing 10% fetal bovine serum to 80% confluence and plated in 60-mm cell culture dishes (1 × 10^6^/ml). Passage 1–2 chondrocytes were pretreated by IL-1β (10 ng/ml) for 24 h to maintain OA pathological condition for the subsequent experiments as described [15–18].

**Table 1:** Information of OA patients with total knee replacement surgery

Age (years)	Case	Sex		Duration of OA (years)		K.L.Image Criterion	
		M	F	≤ 3	≥ 3	III	IV
60–64	13	1	12	6	7	2	11
65–72	14	3	11	2	12	2	12

*K.L.Image Criterion* Kellgren and Lawrence criterion [19]

### Establishment of a rat experimental model of OA

The protocol was approved by the Committee on the Ethics of Animal Experiments of Medical School, Xiamen University. Four- to five-week-old male SD rats (120–150 g) purchased from Slaccas.com (Shanghai, China) were acclimatized to the laboratory environment for one week before the experiments and were randomly divided into three parts, normal (*n* = 12 rats), Sham (*n* = 12 rats), and OA (*n* = 60 rats). Sixty rats underwent anterior cruciate ligament transection and medial menisci resection [15, 17] and were divided into 5 groups, OA (*n* = 12 rats), injection with normal saline (NS, 0.9% NaCl) (*n* = 12 rats, a total volume of 40 μl per injection), injection with IL-1Ra (*n* = 12 rats, 500 μg/ml in a total volume of 40 μl per injection), TAT (*n* = 12 rats, 1.5 mg/kg in a total volume of 40 μl per injection), and IL-1Ra+TAT (*n* = 12 rats, IL-1Ra: 1000 μg/ml in a total volume of 20 μl; TAT: 1.5 mg/kg in a total volume of 20 μl per injection). As described previously [20, 21], different reagents or NS were injected intra-articularly once every three days for two weeks after three days post-surgery. Rats were not sacrificed until three and five weeks after post-operation, respectively. Animal hepatic tissue was collected to detect the side effect of different reagents and NS injected intra-articularly.

### Real-time PCR (RT-PCR)

After total RNA in chondrocytes was extracted using TRIzol (Invitrogen, CA, USA), cDNA was synthesized with 1 μg of total RNA at 37 °C for 15 min using a Primescript RT Master Mix Kit (Takara, Dalian, China). Real-time PCR was then performed using an ABI StepOnePlus Sequence Detection System v2.1 (Applied Biosystems, Singapore) with a SYBR Premix Ex Taq II Kit (Takara, Dalian, China). The results were normalized to GAPDH and analyzed using SDS software v2.1 [22, 23]. The primers used for quantitative PCR to measure gene expression levels are listed in Table 2.

**Table 2:** List of primers in quantitative PCR

Gene	Forward primer	Reverse primer
*ACAN*	5′-TCCGCTGGTCTGATGGACAC-3′	5′-CCAGATCATCACTACGCAGTCCTC-3′
*Col2a1*	5′-TCCTAAGGGTGCCAATGGTGA-3′	5′-GGACCAACTTTGCCTTGAGGAC-3′
*BECN1*	5′-AGCACGCCATGTATAGCAAAGA-3′	5′-GGAAGAGGGAAAGGACAGCAT-3′
*Map1lc3b*	5′-GAGTGGAAGATGTCCGGCTC-3′	5′-CCAGGAGGAAGAAGGCTTGG-3′
*GAPDH*	5′-CAAGTTCAACGGCACAGTCAAG-3′	5′-ACATACTCAGCACCAGCATCAC-3′

### Separation of cytoplasmic and nuclear fraction

Cells were suspended in 2 ml MS buffer (210 mmol/l mannitol, 70 mmol/l sucrose, 5 mmol/l Tris-HCl, pH 7.5, and 1 mmol/l EDTA, pH 7.5), containing a 1% protease inhibitor cocktail, and then homogenized using a Dounce homogenizer [24]. The homogenate was spun at 12,000×*g* for 30 s at 4 °C to pellet the nuclei and unbroken cells. The supernatant was the cytoplasmic fraction.

### Western blotting analysis and immunoprecipitation assay

Protein extracts were subjected to SDS-PAGE (6–12%) and transferred to a PVDF membrane (GE Healthcare, Hertfordshire, UK) as described [22, 23]. The membrane was incubated with various antibodies as required at 4 °C overnight, followed by the addition of the corresponding secondary antibodies at room temperature for 1 to 2 h. An enhanced chemiluminescence (ECL) detection kit was used to detect antibody reactivity (Pierce, Rockford, IL, USA).

As described previously [25, 26], 400 μg of nuclear protein was mixed with 8 μl of Protein A&G Sepharose (Sigma-Aldrich, Shanghai, China) and 8 μl of anti-LC3B antibody or immunoglobulin (IgG) control for 3 h at 4 °C. The protein-antibody complexes that were recovered on the beads were subjected to western blot analysis as above using anti-LC3B and anti-acetylated-lysine antibodies.

### Immunofluorescence assay

Cells were fixed in 4% paraformaldehyde, permeabilized with 0.1% Triton X-100 for 30 min, blocked with 5% BSA, and incubated with anti-LC3B antibody (1:200) overnight, followed by incubation with Cy3-conjugated secondary antibody (1:100) (Boster, Wuhan, China) for 1 h in the dark as described [23, 25]. Nuclei were counterstained with 4′, 6-diamidino-2-phenylindole (DAPI, 50 mg/ml, Sigma) for 1 min. The endogenous LC3B in the stained cells were finally visualized under a fluorescence microscope (Leica Tcs Sp2 SE, Leica, Shanghai, China).

### Transmission electron microscopy

Cells were scraped and then pelleted by centrifugation at 2000×*g* for 15 min at 4 °C, followed by fixation for 2 h at 4 °C in 2.5% glutaraldehyde in 0.1 M PBS (PH7.4) as described [27]. After samples were dehydrated and embedded in Embed-812 resin, 70-nm sections were cut using an ultramicrotome (Leica EM UC7, LEICA, Shanghai, China) and stained with uranyl acetate and lead citrate. Autophagic vacuoles were observed under a transmission electron microscope (Tecnai G2 Spirit BioTWIN, FEI Company, Hillsboro, OR, USA).

### Histopathological assay

Samples were fixed in 4% paraformaldehyde for 48 h followed with decalcification in 10% EDTA-2Na for three weeks, and then paraffin-embedded for further routine histological preparation. Three-micrometer-thick sections were deparaffinized in xylene and rehydrated in graded alcohols and distilled water prior to H.E. and Safranin O-Fast green staining as described [15, 17]. Histological sections were imaged using the Virtual Slide Microscope (VS120-S6-W, Olympus, Tokyo, Japan). The articular cartilage thickness of each femur condyle was measured using Image-Pro Plus 6.0 software. According to the Osteoarthritis Research Society International (OARSI) scoring system established for grading OA changes [28], semi-quantitative histopathological grading was performed by two different blinded pathologists. Score 0 represents normal articular cartilage, and an increasing score indicates a more biologically cartilage degeneration (a maximum possible score of 24).

### Immunohistochemistry staining

Sections were incubated overnight at 4 °C with primary antibody: type II of collagen (Col II, 1:1000), Aggrecan (1:800), autophagy marker LC 3B (1:200), and Beclin1 (1:200) dilutions, respectively, prior to incubation with secondary antibodies, as described in the manufacturer’s instructions (MAIXIN.BIO, Fuzhou, China). Diaminobenzidine was then used to visualize the immunohistochemical reaction followed by being counterstained with hematoxylin. Images were scanned using the Virtual Slide Microscope (VS120-S6-W, Olympus, Tokyo, Japan). Dark brown cells or area were considered to be positive. The positive chondrocytes were counted semi-automated using Image-Pro Plus 6.0 Software, and area was measured using ImageJ Software, followed by analysis with GraphPad Prism version 5 [15, 17, 29, 30].

### Statistical analysis

Data were expressed as the mean ± 95% confidence interval (CI) of three independent experiments in each cell experiment and six independent samples in each group of animal experiments. One-way analysis of variance (ANOVA) with Tukey’s post hoc test was used to compare two or multiple groups by GraphPad Prism 5 software. The error bars of all figures represent 95% CI. A value of *p* < 0.05 was regarded as statistically significant.

## Results

### IL-1 receptor blockade by IL-1Ra restored autophagy to attenuate ECM degradation in rat OA chondrocytes

**Fig. 1:**
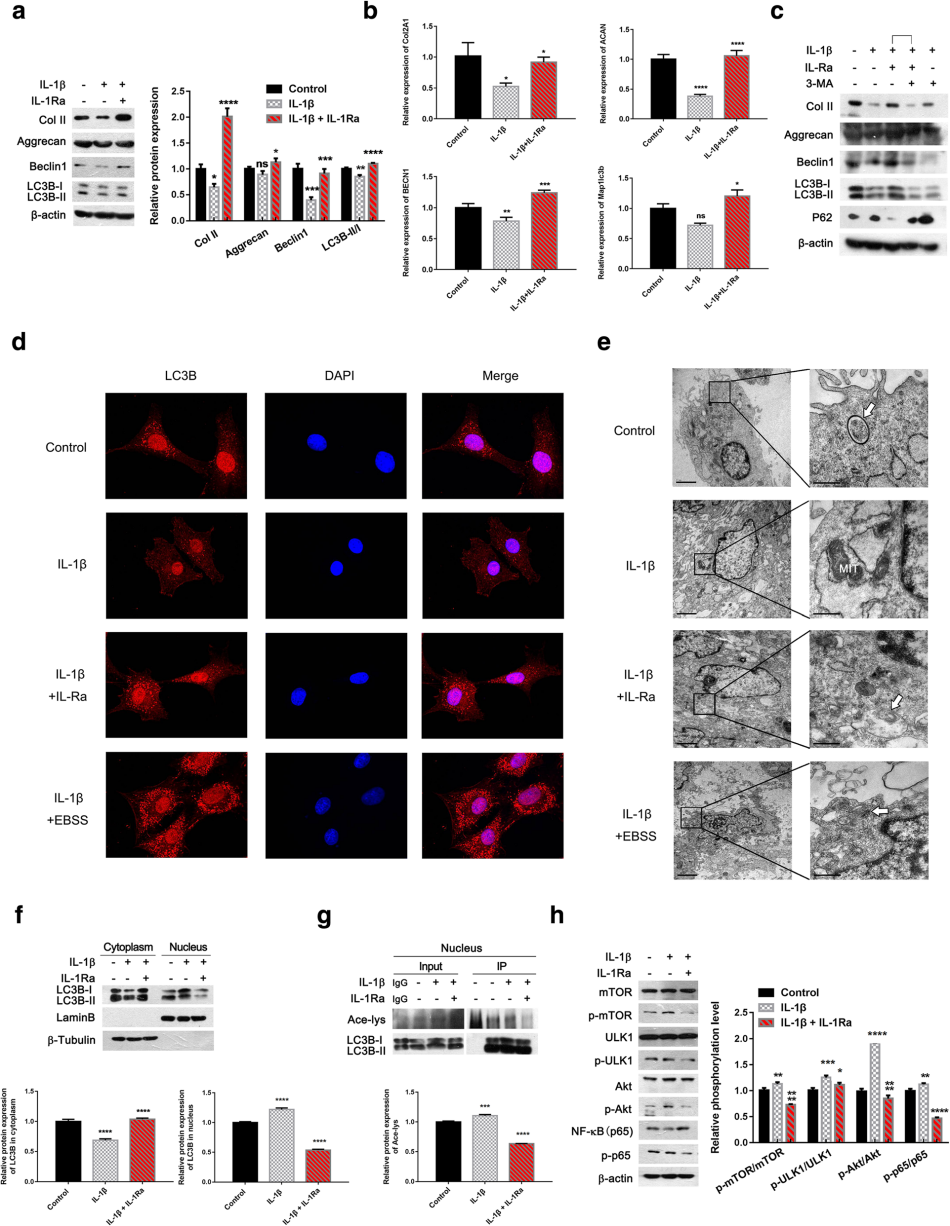
IL-1 receptor blockade by IL-1Ra restored autophagy to attenuate ECM degeneration in rat OA chondrocytes. **a**, **b** Cells pretreated with IL-1β (20 ng/ml) for 36 h were treated with IL-1β (20 ng/ml)+IL-1Ra (40 ng/ml) for 36 h. The Col II (1:2000), Aggrecan (1:1000), Beclin1 (1:1000), LC3B (1:1000), and β-actin (1:40,000) protein levels were detected via western blotting (**a**). Col2A1, AGAN, BECN1, Map1lc3b, and GAPDH mRNA levels were detected via RT-PCR (**b**). **c** Cells pretreated with IL-1β (20 ng/ml) for 36 h were treated with IL-1β (20 ng/ml)+IL-1Ra(40 ng/ml) for 36 h, IL-1β (20 ng/ml) for 33 h followed by co-treatment with 3-MA (10 mM) for 3 h, or IL-1β (20 ng/ml)+3-MA (10 mM) for 3 h followed by co-treatment with IL-1Ra (40 ng/ml) for 33 h, respectively. The Col II (1:2000), Aggrecan (1:1000), Beclin1 (1:1000), LC3B (1:1000), P62 (1:1000), and β-actin (1:40,000) protein levels were detected via western blotting. **d**, **e** Cells pretreated with IL-1β (20 ng/ml) for 36 h were treated with IL-1β (20 ng/ml)+IL-1Ra (40 ng/ml) or IL-1β (20 ng/ml)+EBSS (as positive control) for 36 h, respectively. Representative images of immunofluorescence staining (LC3B (red), nucleus staining-DAPI (blue), original magnification × 40, **d**). Representative images of the transmission electron microscope (autophagic vacuoles indicated by white arrows, **e**). **f**, **g** Cells pretreated with IL-1β (20 ng/ml) for 36 h were treated with IL-1β (20 ng/ml)+IL-1Ra (40 ng/ml) for 36 h, followed by separation of cytoplasmic and nuclear fractions. The LC3B (1:1000), Lamin B (1:1000), and β-tubulin (1:1000) protein levels were detected via western blotting (**f**). Protein extracts in nucleus were subjected to immunoprecipitation with anti-LC3B antibody. The immunoprecipitates were immunoblotted with anti-acetylated-lysine antibody (1:1000) (**g**). **h** Cells pretreated with IL-1β (20 ng/ml) for 36 h were treated with IL-1β (20 ng/ml) + IL-1Ra (40 ng/ml) for 36 h. The mTOR (1:1000), p-mTOR (1:1000), ULK1 (1:1000), p-ULK1 (1:1000), Akt (1:1000), p-Akt (1:1000), NF-κB (p65, 1:1000), p-p65 (1:1000), and β-actin (1:40,000) protein levels were detected via western blotting. The values represent the means ± 95% CI of three independent experiments (*p < 0.05, **p < 0.01, ***p < 0.001, ****p < 0.0001)

In rat chondrocytes, IL-1β significantly reduced Beclin1 protein level and the ratio of LC3B-II/I, along with a significant decrease in Col II protein level, while Aggrecan expression level failed to show a significant difference (Fig. 1a, *p < 0.05, **p < 0.01, ***p < 0.001, vs control chondrocytes). IL-1Ra (an antagonist of IL-1β) significantly reversed the effect of IL-1β on them (Fig. 1a, *p < 0.05, ***p < 0.001, ****p < 0.0001, vs IL-1β-treated chondrocytes). Similarly, IL-1β significantly reduced Col2A1, ACAN, and BECN1 mRNA expression levels, while Map1lc3b level failed to show a significant difference (Fig. 1b, *p < 0.05, **p < 0.01, ****p < 0.0001, vs control chondrocytes), and addition of IL-1Ra significantly reversed the effect of IL-1β on Col1a2, ACAN, BECN1, and Map1lc3b mRNA levels (Fig. 1b, *p < 0.05, ***p < 0.001, ****p < 0.0001, vs IL-1β-treated chondrocytes). Furthermore, the addition of 3-methyladenine (3-MA, an inhibitor of autophagy) attenuated the promoted effect of IL-1Ra on Col II and Aggrecan protein levels in IL-1β-treated chondrocytes (Fig. 1c). Under a fluorescence microscope, the increased LC3B-positive structures (red) were noted in control and IL-1β+IL-1Ra-treated chondrocytes (EBSS as positive control), compared with IL-1β-treated chondrocytes (Fig. 1d). Increased autophagic vacuoles were also observed in IL-1β+IL-1Ra-treated chondrocytes (EBSS as positive control) under an electron microscope, compared with IL-1β-treated chondrocytes (Fig. 1e). Interestingly, the distribution of LC3B-positive structure in nucleus also seemed to be differential between IL-1β-treated and IL-1β+IL-1Ra-treated chondrocytes (Fig. 1d and Additional file 1: Figure S1). In view of the involvement of the acetylation of LC3B in autophagy induction [31–33], we further investigated the distribution and acetylation of LC3B in cytoplasm and nucleus of chondrocytes using separation of cytoplasm and nucleus and immunoprecipitation assay. Figure 1 f shows that IL-1β promoted significantly the translocation of LC3B from cytoplasm to nucleus (****p < 0.0001, vs control chondrocytes), and addition of IL-1Ra significantly reversed the effect of IL-1β on LC3B translocation (Fig. 1f, ****p < 0.0001, vs IL-1β-treated chondrocytes). Figure 1 g shows that IL-1β promoted LC3B acetylation and addition of IL-1Ra significantly led to the deacetylation of LC3B (***p < 0.001, vs control, ****p < 0.0001, vs IL-1β-treated chondrocytes). Additionally, IL-1β significantly upregulated the phosphorylation of mTOR, ULK1, Akt, and NF-κB (p65) (Fig. 1h, **p < 0.01, ***p < 0.001, ****p < 0.0001, vs control chondrocytes). Addition of IL-1Ra significantly reduced the phosphorylation of mTOR, ULK1, Akt, and NF-κB(p65) induced by IL-1β (Fig. 1h,*p < 0.05, ****p < 0.0001, vs IL-1β-treated chondrocytes). Taken together, IL-1β significantly reduced autophagy and IL-1 receptor blockade by IL-1Ra significantly restored autophagy to attenuate ECM degradation in rat IL-1β-treated chondrocytes (mimicking OA chondrocytes), associated with mTOR, ULK1, Akt, NF-κB, and the deacetylation of LC3B.

### IL-1 receptor blockade by IL-1Ra restored autophagy to attenuate ECM degradation in human OA chondrocytes

**Fig. 2:**
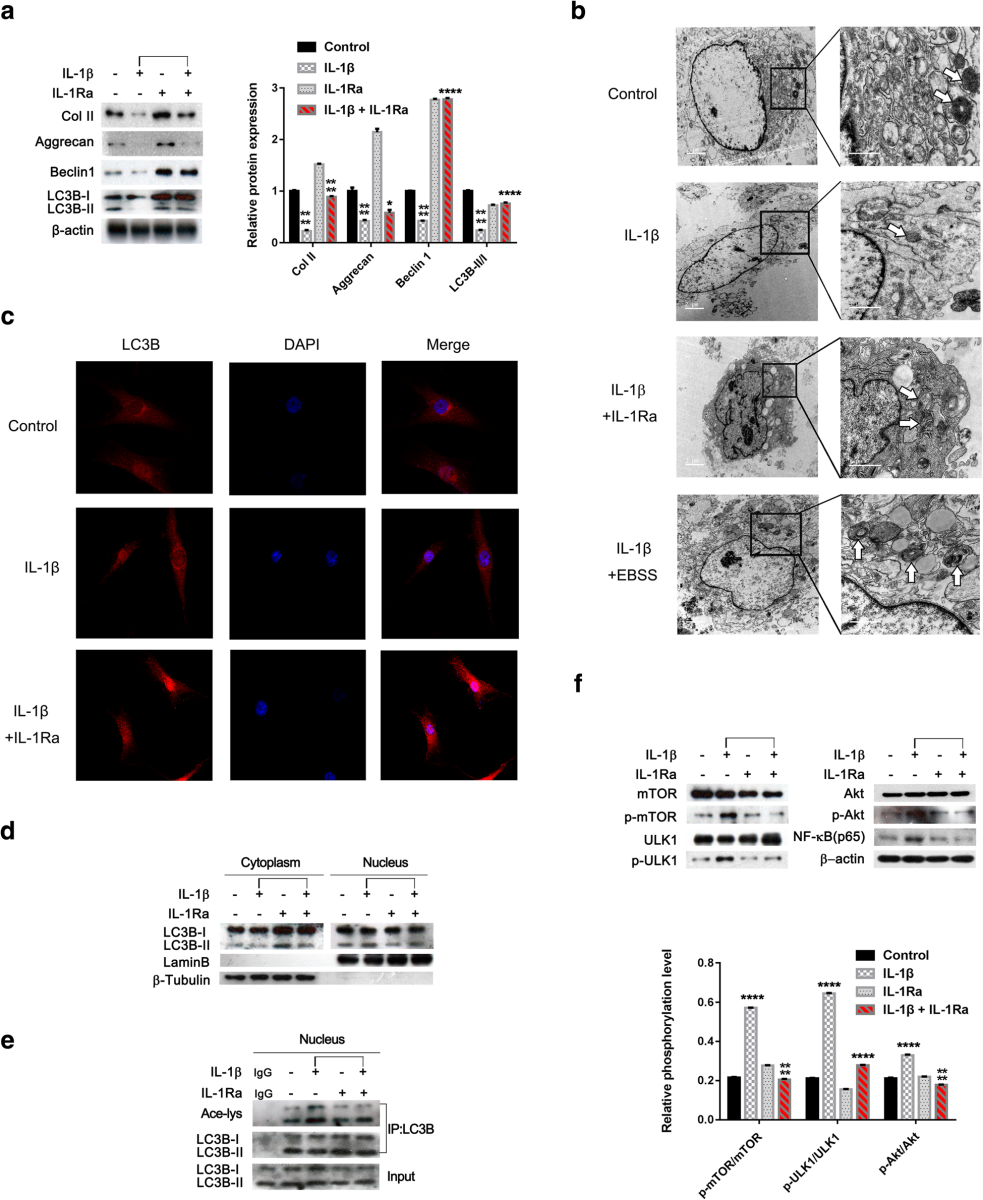
IL-1 receptor blockade by IL-1Ra restored autophagy to attenuate ECM degradation in human OA chondrocytes. **a** Cells were treated with IL-1β (10 ng/ml), IL-1Ra (40 ng/ml), or IL-1β (10 ng/ml)+IL-1Ra (40 ng/ml) for 24 h, respectively. The Col II (1:2000), Aggrecan (1:1000), Beclin1 (1:1000), LC3B (1:1000), and β-actin (1:40,000) protein levels were detected via western blotting. **b** Cells pretreated with IL-1β (10 ng/ml) for 24 h were treated with IL-1β (10 ng/ml)+IL-1Ra (40 ng/ml) or IL-1β (10 ng/ml)+EBSS (as positive control) for 24 h, respectively. Representative images of the transmission electron microscope (autophagic vacuoles indicated by white arrows). **c** Cells pretreated with IL-1β (10 ng/ml) for 24 h were treated with IL-1β (10 ng/ml)+IL-1Ra (40 ng/ml) for 24 h. Cells were treated with or without IL-1Ra (40 ng/ml) and IL-1β (10 ng/ml) for 24 h. Representative images of immunofluorescence staining (LC3B (red) (1:400), nucleus staining-DAPI (blue), original magnification× 40). **d**, **e** Cells were treated with IL-1β (10 ng/ml), IL-1Ra (40 ng/ml), or IL-1β (10 ng/ml)+IL-1Ra (40 ng/ml) for 24 h, respectively, followed by separation of cytoplasmic and nuclear fractions. The LC3B (1:1000), Lamin B (1:1000), and β-tubulin (1:1000) protein levels were detected via western blotting (**d**). Protein extracts in nucleus were subjected to immunoprecipitation with anti-LC3B antibody. The immunoprecipitates were immunoblotted with anti-acetylated-lysine antibody (1:1000) (**e**). **f** Cells were treated with IL-1β (10 ng/ml), IL-1Ra (40 ng/ml), or IL-1β (10 ng/ml)+IL-1Ra (40 ng/ml) for 24 h, respectively. The mTOR (1:1000), p-mTOR (1:1000), ULK1 (1:1000), p-ULK1 (1:1000), Akt (1:1000), p-Akt (1:1000), NF-κB (p65) (1:1000), and β-actin (1:40,000) protein levels were detected via western blotting. Data is representative of three independent experiments

In human OA chondrocytes (Table 1), IL-1β reduced Col II, Aggrecan, Beclin1 protein levels, and the ratio of LC3B-II/I, while addition of IL-1Ra reversed the effect of IL-1β on them (Fig. 2a, *p < 0.05,****p < 0.0001, vs control or IL-1β-treated human OA chondrocytes). Increased autophagic vacuoles were obviously observed in IL-1β+IL-1Ra-treated human OA chondrocytes, compared with IL-1β-treated human chondrocytes (Fig. 2b). LC3B-positive structures in IL-1β+IL-1Ra-treated human OA chondrocytes seemed to be more than that in IL-1β-treated human OA chondrocytes (Fig. 2c). Like rat OA chondrocytes, the differential distribution of LC3B-positive structure in nucleus was observed in human OA chondrocytes between IL-1β-treated and IL-1β+IL-1Ra chondrocytes (Fig. 2c, Additional file 1: Figure S1). The results of the separation of cytoplasmic and nuclear fraction showed that IL-1β promoted the translocation of LC3B from the cytoplasm to nucleus in human OA chondrocytes, and the addition of IL-1Ra reversed the effect of IL-1β on the translocation of LC3B (Fig. 2d). Meanwhile, IL-1Ra led to the deacetylation of LC3B in IL-1β-treated human OA chondrocytes (Fig. 2e). Additionally, IL-1β upregulated the phosphorylation of mTOR, ULK1 and Akt, along with an increase in NF-κB (p65) protein level, and IL-1Ra reversed the effect of IL-1β on them (Fig. 2f, ****p < 0.0001, vs control or IL-1β-treated human OA chondrocytes).

### The effect of IL-1Ra, TAT, and a combination of both on articular cartilage degeneration in a rat OA model

**Fig. 3:**
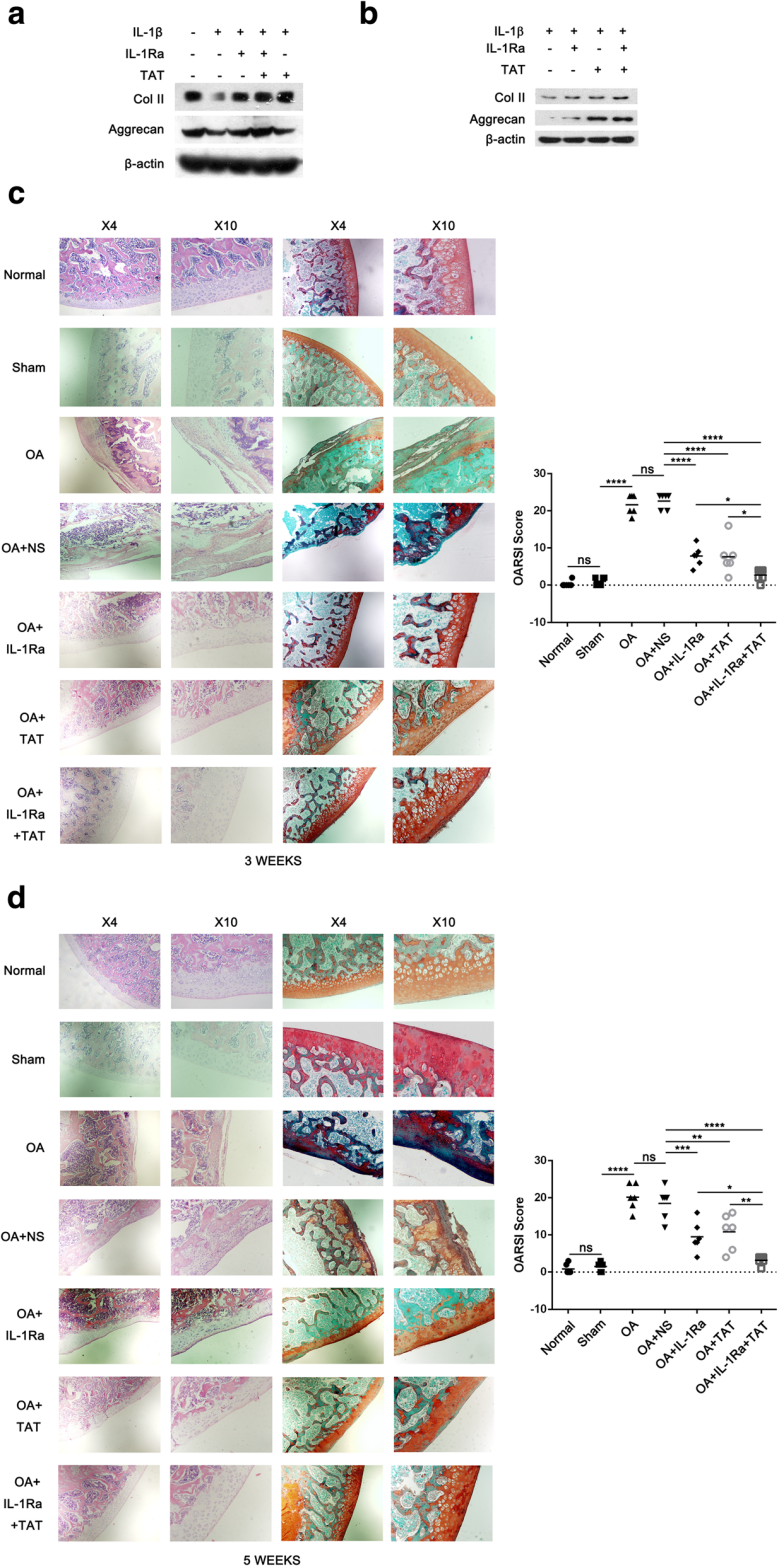
Effect of IL-1Ra, TAT, and IL-1Ra+TAT on articular cartilage degeneration in a rat OA model. **a** Rat chondrocytes pretreated with IL-1β (20 ng/ml) for 36 h were treated by IL-1β (20 ng/ml)+IL-1Ra (40 ng/ml) for 36 h, IL-1β (20 ng/ml) for 28 h followed by co-treatment with TAT (10 μM) for 8 h, or IL-1β (20 ng/ml)+IL-1Ra (40 ng/ml) for 28 h followed by co-treatment with TAT (10 μM) for 8 h, respectively. The Col II (1:2000), Aggrecan (1:1000), and β-actin (1:40,000) protein levels were detected via western blotting. **b** Human OA chondrocytes pretreated with IL-1β (10 ng/ml) for 24 h were treated by IL-1β (10 ng/ml)+IL-1Ra (40 ng/ml) for 24 h, IL-1β (10 ng/ml) for 16 h followed by co-treatment with TAT (10 μM) for 8 h, or IL-1β (10 ng/ml)+IL-1Ra (40 ng/ml) for 16 h followed by co-treatment with TAT (10 μM) for 8 h, respectively. The Col II (1:2000), Aggrecan (1:1000), and β-actin (1:40,000) protein levels were detected via western blotting. **c**, **d** IL-1Ra (500 μg/ml, 40 μl), TAT (1.5 mg/kg), and NS (normal saline, 0.9% NaCl) were injected intra-articularly in the knees of a rat OA model once every three days for two weeks, respectively. Representative images (left) of H.E. and Safranin O-Fast green staining and graphs (right) of the OARSI scores in three weeks post-operative groups (**c**). Representative images (left) of H.E. and Safranin O-Fast green staining and graphs (right) of the OARSI scores in five weeks post-operative groups (**d**). The values represent the means ± 95% CI (*n* = 6,*p < 0.05, *p < 0.05, **p < 0.01, ****p < 0.0001, original magnification× 4, × 10)

Western blotting analysis showed that the promoted effect of a combination of IL-1Ra with TAT-Beclin1 (autophagy inducer, TAT) on Col II and Aggrecan protein levels was more effective than IL-1Ra alone in IL-1β-treated rat and human OA chondrocytes (Fig. 3a, b). Hence, we investigated the effect of IL-1Ra, TAT, and a combination of both on cartilage degeneration and whether the “additive” or “synergistic” effect of IL-1Ra and TAT treatment exists in rat OA model. Each knee was cut through the intercondylar of femur, and femoral cartilage was then observed in the current study as tibial cartilage was not intact at some sections (Additional file 2 Figure S2 and Additional file 3: Figure S3). The intra-articular injection of IL-1Ra, TAT, or IL-1Ra+TAT in three weeks post-operative groups resulted in a significant decrease of OARSI score (Fig. 3c, ****p < 0.0001, vs OA+NS group). Similar results were observed in five weeks post-operative groups (Fig. 3d, **p < 0.01, ****p < 0.0001, vs OA+NS group). In either three weeks or five weeks post-operative groups, OARSI score of IL-1Ra+TAT group was lower than IL-1Ra or TAT alone (Fig. 3c,d, *p < 0.05,**p < 0.01), but closer to that of the normal group. Additionally, the intra-articular injection of IL-1Ra and TAT did not result in liver injury of rat OA model (Additional file 4: Figure S4).

### The effect of IL-1Ra, TAT, and a combination of both on matrix synthesis and autophagy in articular cartilage of a rat OA model

**Fig. 4:**
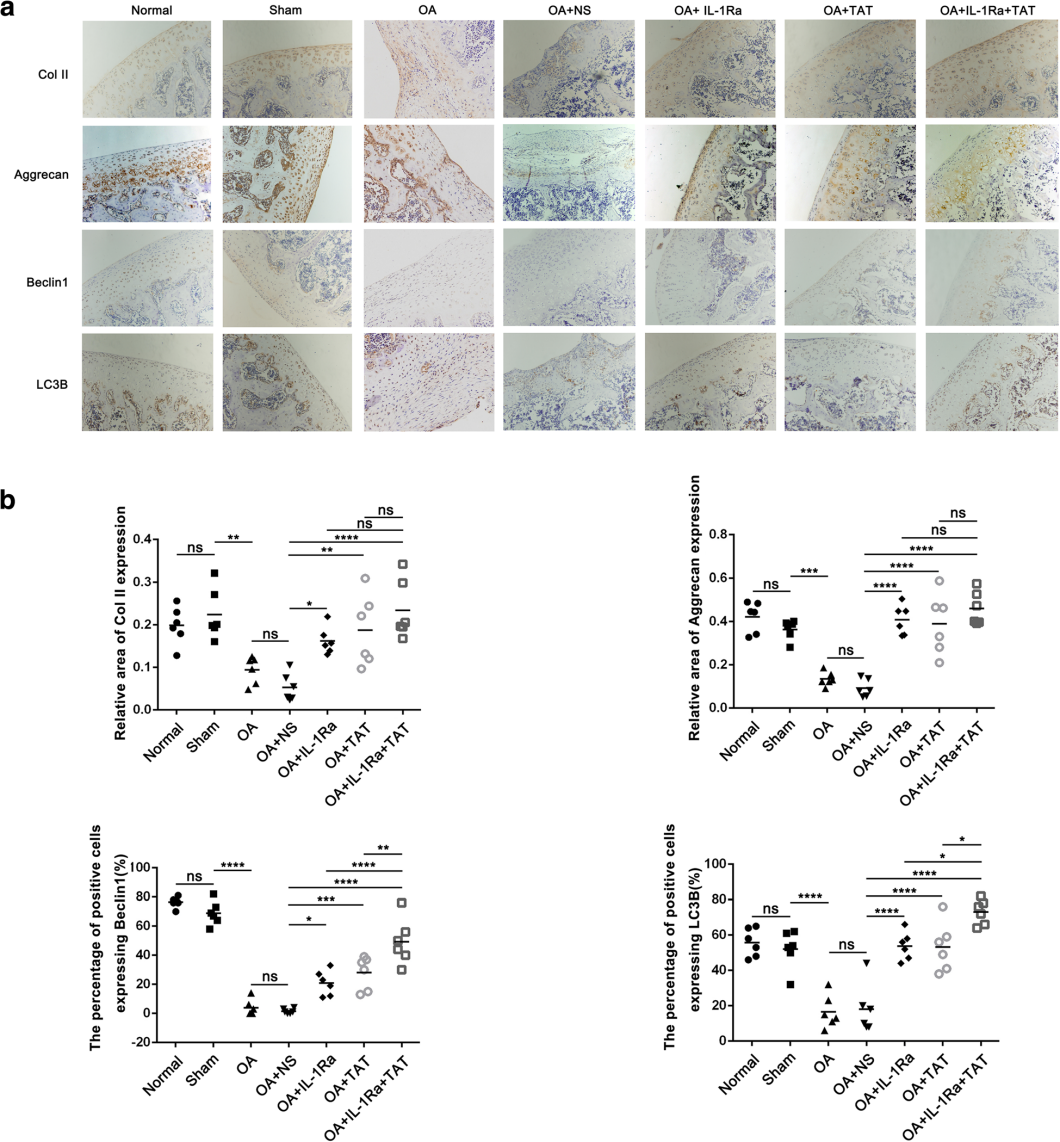
Effect of IL-1Ra, TAT, and IL-1Ra+TAT on ECM and autophagy in three-week-post-operative OA rats. **a** Representative images of immunohistochemistry staining for Col II (1:200), Aggrecan (1:200), Beclin1 (1:200), and LC3B (1:200) (original magnification × 10). **b** Graphs indicating the relative area of Col II and Aggrecan expression and the relative positive cells of Beclin1 and LC3B. The values represent the means ± 95% CI (**p < 0.01, ***p < 0.001, ****p < 0.0001)

**Fig. 5:**
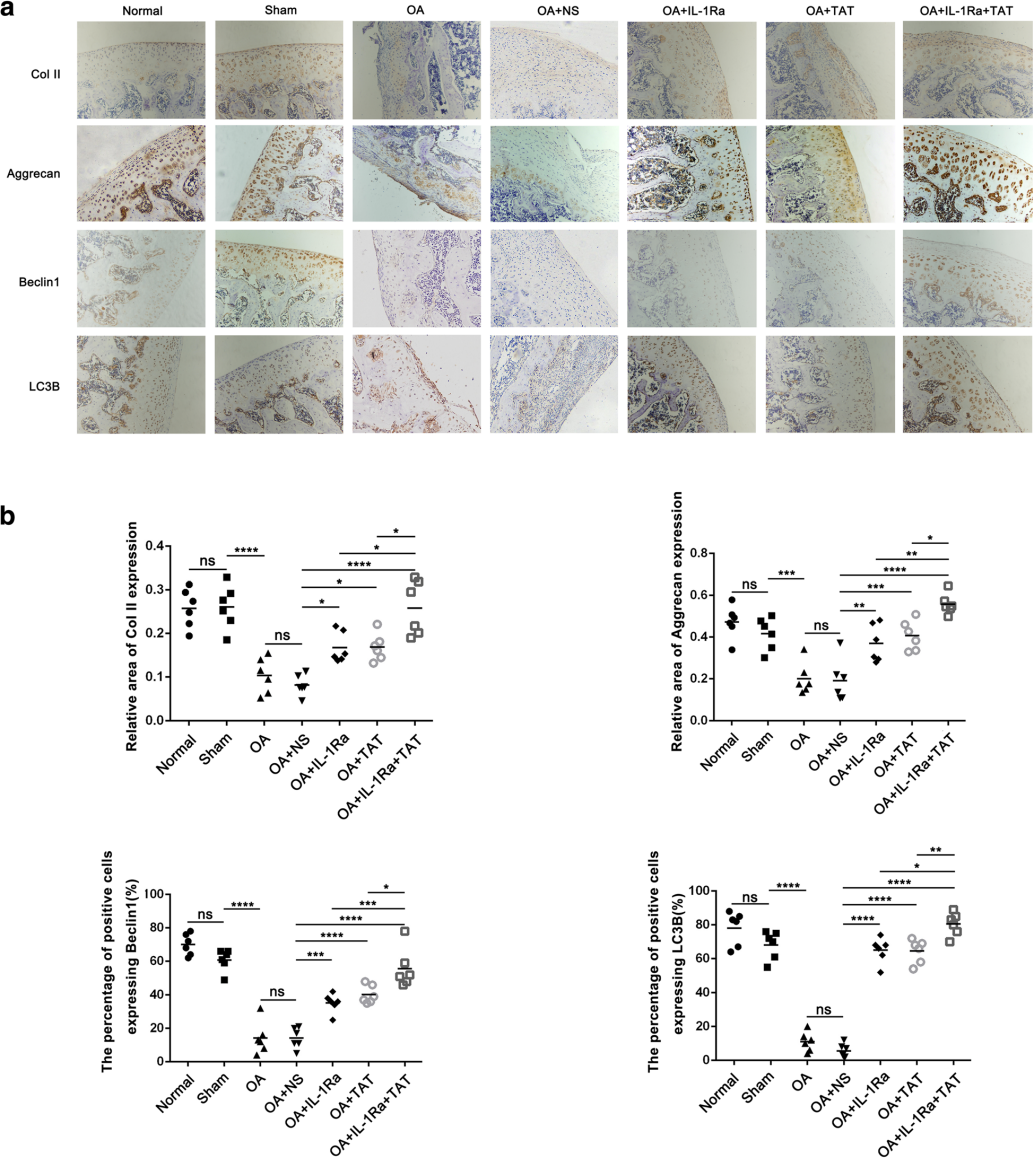
Effect of IL-1Ra, TAT, and IL-1Ra+TAT on ECM and autophagy in five-week-post-operative OA rats. **a** Representative images of immunohistochemistry staining for Col II (1:200), Aggrecan (1:200), Beclin1 (1:200), and LC3B (1:200) (original magnification × 10). **b** Graphs indicating the relative areas of Col II and Aggrecan expression and the relative positive cells of Beclin1 and LC3B. The values represent the means ± 95% CI (**p < 0.01, ***p < 0.001, ****p < 0.0001)

In three weeks post-operative groups, the relative area of Col II and Aggrecan protein expression increased significantly in IL-1Ra-, TAT-, and IL-1Ra+TAT-treated groups (Fig. 4, *p < 0.05,**p < 0.01,****p < 0.0001, vs OA+NS group). The relative area of both Col II and Aggrecan protein expression in IL-1Ra+TAT-treated groups was not significantly higher than IL-1Ra or TAT alone (Fig. 4). The percentage of positive cells expressing Beclin1 or LC3B increased significantly in IL-1Ra-, TAT-, and IL-1Ra+TAT-treated groups (Fig. 4, *p < 0.05, ***p < 0.001, ****p < 0.0001, vs OA+NS group), while the percentage of positive cells expressing both Beclin1 or LC3B in IL-1Ra+TAT-treated groups was also significantly higher than IL-1Ra or TAT alone (Fig. 4, *p < 0.05, **p < 0.01, ***p < 0.001). In five weeks post-operative groups, the relative area of Col II and Aggrecan protein expression also increased significantly in IL-1Ra-, TAT-, and IL-1Ra+TAT-treated groups (Fig. 5, *p < 0.05, **p < 0.01, ***p < 0.001, ****p < 0.0001, vs OA+NS group). The relative area of Col II and Aggrecan protein expression in IL-1Ra+TAT-treated group was significantly higher than IL-1Ra or TAT alone (Fig. 5, *p < 0.05, **p < 0.01). Similar to three weeks post-operative groups, the percentage of positive cells expressing Beclin1 or LC3B increased significantly in IL-1Ra-, TAT-, and IL-1Ra+TAT-treated groups (Fig. 5, ***p < 0.001, ****p < 0.0001, vs NS group), and the percentage of positive cells expressing both Beclin1 or LC3B in IL-1Ra+TAT-treated groups was significantly higher than IL-1Ra or TAT alone (Fig. 5, *p < 0.01, **p < 0.01, ***p < 0.001).

## Discussion

Our findings demonstrated that IL-1β degraded ECM synthesis concomitant with reduced autophagy in IL-1β-treated rat and human OA chondrocytes. IL-1Ra reversed the effect of IL-1β by restoring autophagy, associated with Akt/mTOR/ULK1 and Akt/NF-κB signaling pathways, as well as the deacetylation of LC3B. Furthermore, intra-articular injection of IL-1Ra, TAT, and IL-1Ra+TAT, respectively, ameliorated cartilage degeneration in a rat OA model, in which the effect of a combination of IL-1Ra with TAT was significantly higher than IL-1Ra alone. Therefore, reduced autophagy could contribute to ECM degradation induced by IL-1β and IL-1 receptor blockade by IL-1Ra could restore autophagy to promote ECM synthesis in OA chondrocytes in vitro and in vivo. A combination of blocking IL-1β effect by IL-1 receptor antagonist with enhancing autophagy induction by autophagy inducer (TAT) was more effective than IL-1Ra alone, bringing a new insight into therapeutic strategy for OA.

Previous studies have shown that the autophagy induction suppresses IL-1β secretion and IL-1 receptor blockade could restore autophagy in macrophages [8–10]. Consistent with those studies, in the current study, IL-1β degraded ECM synthesis concomitant with reduced autophagy and IL-1Ra reversed its effect by enhancing autophagy induction in rat and human OA chondrocytes, while 3-MA reversed the effect of IL-1Ra. Furthermore, in rat OA model, intra-articular injection of IL-1Ra ameliorated cartilage degeneration, similar to an autophagy inducer TAT that could protect mammalian cells against damage by autophagy induction [34–36]. Hence, we suggested that reduced autophagy could contribute to ECM degradation induced by IL-1β and that IL-1 receptor blockade by IL-1Ra could restore autophagy to promote ECM synthesis in OA chondrocytes in vitro and in vivo. As for why Col II and Aggrecan predominantly were distributed in the intra- and pericellular region in this study, there might exist two possibilities. One might be associated with the duration and dose of injected reagents. Another might be due to the shortage of detecting effectiveness. This will be considered in the future work.

mTOR, as a “classical” autophagy suppressor, acts by blocking the activity of the ULK1 complex [37]. Akt, an important signal molecule in cell events, has been reported to activate mTOR pathway to suppress autophagy [38, 39]. For instance, microRNA-99 family modulates hepatitis B virus replication by promoting IGF-1R/PI3K/Akt/mTOR/ULK1 signaling-induced autophagy [38] PRKCD/Akt/mTOR/ULK1 signaling pathway is involved in autophagy suppression in cisplatin nephrotoxicity [39]. In the current study, IL-1Ra restored autophagy reduced by IL-1β and reversed the promoted effect of IL1β on the phosphorylation of Akt, mTOR, and ULK1, implying that Akt/mTOR/ULK1 pathway could be involved in OA chondrocyte autophagy restored by IL-1Ra, consistent with the abovementioned studies. In addition, in the current study, IL-1Ra led to the decrease of NF-κB, one of the downstreams of Akt, in rat and human OA chondrocytes. Li et al. have found that cerebral ischemia induced autophagy-like injury is regulated by the NF-κB pathway [40]. Huang et al. have recently reported that the inhibition of the mTOR/NF-κB signaling pathway potentiates HTEA against myocardial IR injury by autophagy and apoptosis in rats [41]. It is believable that the Akt/mTOR/NF-κB pathway was also involved in IL-1Ra-restored autophagy in OA chondrocytes. Therefore, we suggested that IL-1Ra could restore autophagy via Akt/mTOR/ULK1 and Akt/mTOR/NF-κB pathways in rat and human OA chondrocytes.

Differential acetylation of autophagy-related proteins participates in autophagic flux. For instance, LC3B-II deacetylation by HDAC6 is involved in serum-starvation-induced autophagic degradation of Hela cells [31]. Erythropoietin (EPO) alleviates hepatic steatosis by activating autophagy via SIRT1-dependent deacetylation of LC3 [32]. In response to nutrient depletion, activated Sirt1 interacts with and deacetylates nuclear LC3. Through binding to DOR, deacetylated LC3 is transported to the cytoplasm to carry out PE conjugation by sequential interaction with Atg7 and Atg3 [33]. Consistent with these studies, we also found that IL-1Ra led to the translocation of LC3B from nucleus to cytoplasm via its deacetylation, suggesting that LC3B deacetylation could contribute to the restoration of autophagy induction by IL-1Ra in rat and human OA chondrocytes. The regulatory mechanism of LC3B deacetylation in OA pathological progression is further to be studied.

Recently, Elsaid KA et al. reported that IL-1 Ra combined with recombinant human lubricin (rhPRG4) may act synergistically to reduce cartilage catabolism [20], hinting that a combined treatment of IL-1Ra with other components to regulate cartilage metabolism may be more effective than IL-1Ra alone. We found that IL-1Ra combined with TAT ameliorated cartilage degeneration significantly, closer to the normal groups in a rat OA model, even if Col II and Aggrecan expression area of the IL-1Ra+TAT group in three weeks post-operative groups did not change significantly, compared with IL-1Ra or TAT group alone. Hence, we predict that a combined treatment of IL-1 receptor blockade with autophagy enhancement may act synergistically to ameliorate cartilage degeneration of OA. More experiments are needed to prove this hypothesis.

## Conclusions

IL-1Ra restored autophagy and attenuated ECM degradation, in which Akt/mTOR/ULK1, Akt/mTOR/NF-κB, and LC3B deacetylation were involved in rat and human OA chondrocytes, as well as a rat OA model. IL-1β receptor antagonist (IL-1Ra) combined with an autophagy inducer (TAT-Beclin1) is an effective alternative for attenuating extracellular matrix degradation of osteoarthritis in rats and humans.

## Additional files

Additional file 1: **Figure S1.** Column graph of LC3B expression in cytoplasm of rat and human chondrocytes. Relative positive cells of LC3B expression in cytoplasm of rat and human chondrocytes treated with IL-1β or IL-1β+IL-1Ra. (TIF 96 kb)

Additional file 2: **Figure S2.** Samples site images in 3-week-operative OA rats. Under light microscope, sagittal sections with Safranin O-Fast green staining in rat OA model injected with injected with different agents, respectively, were observed (original magnification × 2, × 4). (JPG 7808 kb)

Additional file 3: **Figure S3.** Samples site images in 5-week-operative OA rats. Under light microscope, sagittal sections with Safranin O-Fast green staining in rat OA model injected with injected with different agents, respectively, were observed (original magnification × 2, × 4). (JPG 8389 kb)

Additional file 4: **Figure S4.** Morphological changes of liver in a rat OA model. Under light microscope, livers in normal and OA model injected with IL-1Ra and TAT, respectively, were observed (original magnification × 10). (TIF 608 kb)

## Data Availability

The datasets analyzed during the current study are available from the corresponding author on reasonable request.

## Abbreviations

3-MA: 3-Methyladenine
Ace-lys: Acetylated-lysine antibody
Agg: Aggrecan
Akt: Protein kinase B
Col II: Type II of collagen
ECM: Extracellular matrix
IL-1Ra: Interleukin-1 receptor antagonist
IL-1β: Interleukin-1β
LC3B: Autophagy marker light chain 3B
mTOR: Mammalian target of rapamycin
NF-κB: Transcription factors of the Nuclear Factor κB
NS: Normal saline (0.9% NaCl)
OA: Osteoarthritis
OARSI: Osteoarthritis Research Society International
SD: Sprague–Dawley
ULK1: UNC-51-like kinase 1
