# Supplementary figures and images for: IL-1β receptor antagonist (IL-1Ra) combined with autophagy inducer (TAT-Beclin1) is an effective alternative for attenuating extracellular matrix degradation in rat and human osteoarthritis chondrocytes
Source: Arthritis Res Ther. 2019 Jul 10;21:171. doi: 10.1186/s13075-019-1952-5 (PMC6617669; doi:10.1186/s13075-019-1952-5)

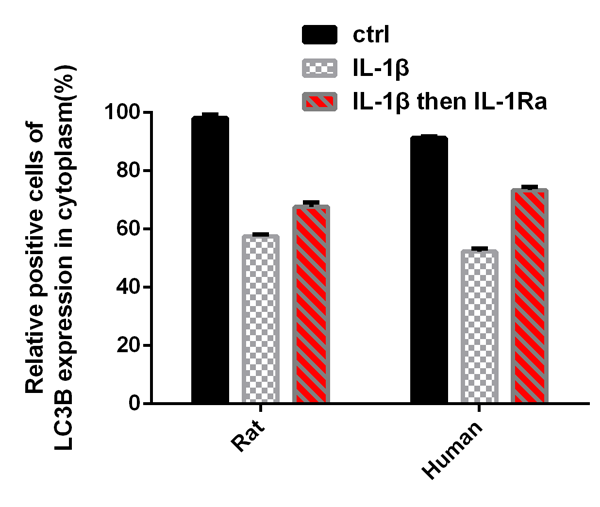

Supplement: Supplementary file 1 — Figure S1. Column graph of LC3B expression in cytoplasm of rat and human chondrocytes. Relative positive cells of LC3B expression in cytoplasm of rat and human chondrocytes treated with IL-1β or IL-1β+IL-1Ra. (TIF 96 kb) [file 13075_2019_1952_MOESM1_ESM.tif]

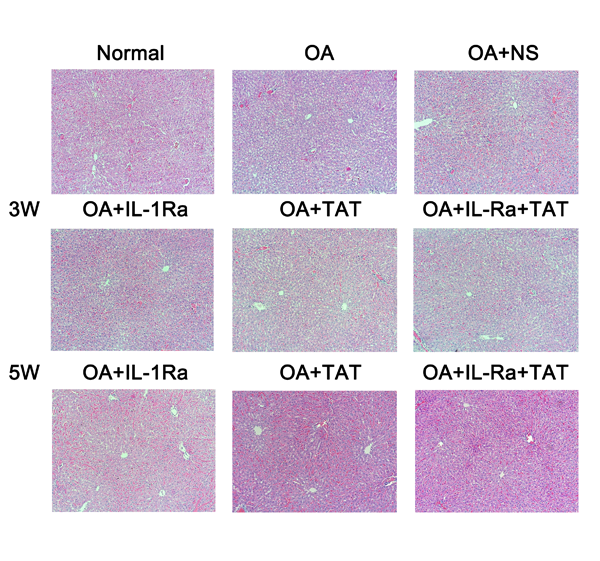

Supplement: Supplementary file 4 — Figure S4. Morphological changes of liver in a rat OA model. Under light microscope, livers in normal and OA model injected with IL-1Ra and TAT, respectively, were observed (original magnification × 10). (TIF 608 kb) [file 13075_2019_1952_MOESM4_ESM.tif]
